# Body Weight Dissatisfaction Is Associated with Cardiovascular Health-Risk Behaviors among Brazilian Adolescents: Findings from a National Survey

**DOI:** 10.3390/ijerph17238929

**Published:** 2020-12-01

**Authors:** Luciane Duarte, Elizabeth Fujimori, Ana Luiza Borges, Aline Kurihayashi, Mary Steen, Alejandra Roman Lay

**Affiliations:** 1 Chronic Noncommunicable Diseases Division, Disease Control Coordination, São Paulo State Health Department, São Paulo 01246-000, Brazil; 2 Public Health Nursing Department, School of Nursing, University of São Paulo, São Paulo 05403-000, Brazil; efujimor@usp.br (E.F.); alvilela@usp.br (A.L.B.); alineyukari@usp.br (A.K.); 3 Clinical and Health Sciences, University of South Australia, Adelaide, SA 5001, Australia; mary.steen@unisa.edu.au; 4 Faculty of Health Sciences, University of Tarapacá, Arica 1000000, Chile; alejandra.roman.lay@gmail.com

**Keywords:** adolescence, body weight dissatisfaction, body image, alcohol drinking, tobacco use, exercise, breakfast, cardiovascular diseases

## Abstract

Body weight dissatisfaction (BWD) among adolescents may be a predictor of adoption of health-risk behaviors. The study aimed to assess the gendered association between two forms of BWD (feeling underweight/overweight) and cardiovascular health-risk behaviors among Brazilian adolescents. This cross-sectional study used data from the National Study of Cardiovascular Risks in Adolescents (ERICA) in Brazil, including 71,740 adolescents aged 12–17 years. BWD was defined as satisfied, dissatisfied feeling underweight and dissatisfied feeling overweight. We considered four health-risk behaviors: tobacco use, alcohol use, physical inactivity and skipping breakfast. Assessment of the associations between BWD and these behaviors were undertaken using logistic regression models. All analyses were stratified by gender. Analyses revealed that 14.9% of male adolescents and 14.5% of female adolescents were dissatisfied feeling underweight and 21.5% of males and 39.9% of females were dissatisfied feeling overweight. Among male adolescents, dissatisfied feeling overweight was associated with greater odds of physical inactivity and skipping breakfast. Among female adolescents, dissatisfied feeling underweight and overweight were associated with higher odds of alcohol use and skipping breakfast. These results highlight the importance of BWD and the association with a range of adolescent health behaviors increasing cardiovascular risk over the life course, depending on gender.

## 1. Introduction

Globally, cardiovascular diseases are responsible for 46% of deaths across all age groups [1]. The World Health Organization (WHO) has identified four major behaviors that contribute to this important noncommunicable disease (NCD), specifically: unhealthy diet, physical inactivity, tobacco use and harmful use of alcohol [1]. These preventable behaviors usually emerge in the foundational years of adolescence that shape health trajectories over the life course. Thus adolescence is seen as a critical window of opportunity to promote healthy lifestyles, with prolonged effects in adult life [2].

It is also acknowledged that, during adolescence, young people reinterpret their self-image in relation to physical and cognitive changes [3] (pp. 129–137). Some studies among adolescents have shown an association between body image and health behaviors related to cardiovascular risks [4,5,6].

A number of researchers have examined young people’s assessment of their body image, and have proposed a framework defining body concept as a multidimensional construct comprising the perceptions (perceptual dimension) and attitudes (attitudinal dimensions) one holds about one’s personal body [3] (pp. 129–137). Specifically, the perceptual dimension refers to an individual’s self-perception of the appearance, while the attitudinal dimension is subdivided into four components: affective, cognitive, behavioral and satisfaction. The affective component includes feelings related to one’s appearance; the cognitive component refers to knowledge about body image; the behavioral component considers body-checking behaviors and the actions to avoid situations or objects that evoke body image concerns; and the satisfaction component relates to person’s appreciation over the body as a whole or to specific parts [3] (pp. 129–137).

Within this framework, body weight satisfaction or dissatisfaction stands out as an important component that needs to be considered in any health assessment, because this dynamic physical marker is used to form and develop body self-assessment [3] (pp. 129–137). However, the definition of body weight dissatisfaction (BWD) varies across studies: some focus on BWD as feelings related to one’s appearance, while others focus on appearance of body weight, or, even, on behaviors to change weight [3] (pp. 129–137). Therefore, due to this range of measures, studies about BWD and its association with health behaviors related to cardiovascular risk are needed.

The Health Behavior in School-aged Children (HBSC) 2013/2014 study has shown high rates of BWD, averaging 50% [7]. Girls are generally more likely to express weight-related dissatisfaction than boys. Likewise, overweight adolescents are more likely to report weight dissatisfaction than normal or underweight adolescents [7]. However, BWD can result from dissatisfied feeling underweight as well as dissatisfied feeling overweight, which is reported by adolescents who believe that they are either underweight or overweight [7]. Therefore, BWD can be divided into two forms: dissatisfied feeling underweight and dissatisfied feeling overweight, which are likely to have different implications in terms of health-related behaviors relating to cardiovascular diseases. In addition, gendered attitudes towards body weight are likely to have a different impact upon cardiovascular diseases related pathways for boys and girls. However, such analyses have not been addressed in the literature [8,9,10,11,12,13].

In Brazil, the setting of this study, overall BWD ranges from 20% to 60% among adolescents [14,15]. However, few studies have investigated the association between BWD and behaviors related to cardiovascular risk in Brazilian adolescents, such as tobacco use [9], alcohol use [9] and physical inactivity [16,17]. In addition, no study has considered the two forms of BWD or the skipping of breakfast as a health-risk behavior. This analysis is significant, since these behaviors are prevalent among Brazilian adolescents. Adolescents aged 13 to 15 and 16 to 17, respectively, declared: 35% and 38% skipped breakfast; 34% and 43% are inactive; 5% and 8% smoked cigarettes in the last 30 days; and 24% and 38% drank alcohol in the last 30 days [18].

This study will address these identified gaps by evaluating the gendered associations between two forms of BWD and a range of health behaviors increasing cardiovascular diseases. Therefore, the aim of the present study was to assess the gendered association between two forms of BWD (feeling underweight/overweight) and cardiovascular health-risk behaviors among Brazilian adolescents. The researchers hypothesize that having a dissatisfied feeling of being underweight or overweight will show different implications for health behaviors and that these effects will vary by gender.

## 2. Materials and Methods

### 2.1. Study Design and Population

Data were derived from the Study of Cardiovascular Risks in Adolescents (ERICA), a national, school-based cross-sectional study, undertaken in Brazil in 2014. The goal of ERICA was to estimate the prevalence of cardiovascular risk factors among adolescent aged 12 to 17 years living in cities of more than 100,000 inhabitants in Brazil. ERICA was approved by the Committee for Ethics in Research (CEP) of the Institute of Collective Health Studies, Universidade Federal do Rio de Janeiro (Process 45/2008) and by a CEP of each unit of the Federation.

The survey used a two-stage strategy. Firstly, 32 geographic strata were selected (27 capitals of each Brazilian state, the federal district and five macroregions of the country), to be representative of large cities at the national level (capitals of each Brazilian state and the federal district) and at the regional level (macroregions of the country). After geographic stratification, two successive selection stages were implemented: selection of schools and selection of school classes. Regarding the selection of schools, 1247 schools were selected based on the number of students in eligible classes and the distance from the state capital. Using class year as a proxy of age, only the classes of seventh (adolescents aged 12 years), eighth (adolescents aged 13 years) and ninth (adolescents aged 14 years) years of elementary school; and the first (adolescents aged 15 years), second (adolescents aged 16 years) and third (adolescents aged 17 years) years of high school were eligible for selection. The classes were selected with different combinations of scheduled time at school (morning and afternoon). Sample design and protocol details are described elsewhere [19]. Students from the selected classes were invited to participate in the research. Written informed consent was obtained from parents and students. The inclusion criteria were adolescents who were aged 12 to 17 years. The exclusion criteria were pregnant girls, or individuals with a physical or mental disability [19].

For the calculation of sample size, a prevalence of the metabolic syndrome in adolescents of 4% was used with a maximum absolute error of 1% and confidence level of 95%. This represents a simple random sample size of 1475 adolescents. However, considering that the sample is clustered by school and class, a design effect of three was used, which in turn has demonstrated a sample size of 4425 adolescents. As the estimates with controlled precision had to be produced for 12 domains (six ages × two genders), it was estimated that the overall sample size would have to be 74,340 individuals [19].

The final sample of the ERICA study consisted of 74,589 participants, however, we assessed data of 71,740 adolescents (36,013 males and 35,727 females) aged 12 to 17 years, which contained complete information about BWD and health-risk behavior.

Adolescents self-completed a questionnaire using an electronic data-collecting personal digital assistant. The questionnaire was partially based on instruments used in other studies on risk factors in the young in Brazil [19]. In addition, trained evaluators, according to written standardized procedures, assessed adolescent’s anthropometric measures (weight and height). These evaluators were healthcare workers, nurses and physical educators. P200M Líder^®^ scale and a portable and collapsible Alturaexata^®^ stadiometer were used [19].

### 2.2. Measures

In this current analysis, the key independent variable was BWD defined in three categories: satisfied, dissatisfied feeling underweight and dissatisfied feeling overweight. The measure was assessed by the following question: “Are you satisfied with your own body weight?” with response options, “Yes, I am satisfied”, “No, I am dissatisfied feeling underweight”, or “No, I am dissatisfied feeling overweight”.

The outcome measures of health behaviors related to cardiovascular risk included tobacco use, alcohol use, physical inactivity and skipping breakfast. Both tobacco and alcohol use were dichotomous measures based on reports of smoking at least one cigarette or alcohol consumption in the past 30 days. Physical inactivity was defined as practicing less than 60 min of moderate- to vigorous-intensity physical activity (MVPA) daily (dichotomous) [20]. Duration of MVPA was assessed using a checklist, which included 24 activities (relating to leisure time and commuting). Adolescents reported on the frequency (days) and the duration (hours and minutes) of each activity in the last seven days. The MVPA questionnaire has been previously shown to have acceptable reliability and validity among Brazilian adolescents [21]. Daily physical inactivity was calculated by summing up the time spent in each activity across the last seven days and divided by seven to obtain a daily average; the measure was dichotomized based on the 60 min per day threshold. Finally skipping breakfast was defined based on common daily consumption of breakfast. Adolescents who reported having breakfast sometimes or never were classified as skipping breakfast and those who ate breakfast every day or almost every day were not.

Information was also collected about adolescents’ individual and sociodemographic characteristics: age group (12–14 years/15–17 years); gender (male/female); skin color (white/nonwhite (black/brown/yellow/indigenous)); mother’s schooling (<11 years of schooling/≥11 years of schooling); family structure (mother and father/only mother/only father or no parents); school type (public/private) and weight status (underweight/adequate weight/overweight or obesity). Adolescents’ weight status was based on anthropometric measures and classified using the Body Mass Index (BMI)-for-age according to reference curves from the WHO guidance, which are specified by gender [22]. The following cut-off points were adopted: Z-score < −2 sd (underweight); Z-score ≥ −2 sd and ≤ +1 sd (adequate weight); Z-score > +1 sd and ≤ +2 sd (overweight); Z-score > +2 sd (obesity). In addition, the school type was considered as a proxy for socioeconomic status, as adolescents who attend private schools show higher scores of Economic Social and Cultural Status in Brazil [23].

### 2.3. Statistical Analysis

Firstly, to describe the study sample descriptive statistics (Chi-square test) was used to characterize bivariate associations between BWD and adolescent’s individual and sociodemographics characteristics as well as their health behaviors. To further evaluate the associations between the two forms of BWD and health-risk behaviors, multivariate logistic regression models were utilized, with adjustments for potential confounders: age, BMI-for-age and school type. For all regression analyses, relevant interaction terms between BWD and age and BMI-for-age were examined. The interaction terms with BMI-for-age were significant, thus further analyses were conducted within each BMI-for-age category separately. The results of the logistic regression are presented as odds ratio (OR) and 95% confidence interval (CI). All analyses were conducted using STATA (Stata Corp LP, College Station, TX, USA), using sample weights to account for the complex survey design.

## 3. Results

### 3.1. Characteristics of the Sample

Adolescents were equally split by gender and half of the sample was 12–14 years (52.7%). The majority was nonwhite (60.0%) and over a half were living with both parents (57.3%), and had mothers with 11 years or more of schooling (55.1%). The majority of adolescents were studying in public schools (82.6%). Seven in ten adolescents had an age appropriate body mass index (71.8%), but 45% reported BWD, mostly in the category of feeling overweight (30.3%). Moreover, BWD was more common amongst girls than boys (53.8% versus 36.4%, *p* < 0.001), in particular with respect of dissatisfaction feeling overweight (39.3% of girls versus 21.5% of boys) (Table 1).

A small percentage of adolescents reported tobacco use (4.8%) and about one fifth reported alcohol use (22.1%). Almost half (48.4%) were inactive. Overall, one fifth (21.9%) reported skipping breakfast. Girls were more likely to be categorized as inactive and were more likely to skip breakfast than boys (Table 1).

Female adolescents were more dissatisfied than male adolescents, and were especially dissatisfied feeling overweight, if they were 15–17 years, white, with mothers that had 11 years or more of schooling, and studying in private schools, and had underweight status or overweight status (Table 2).

### 3.2. Associations between Two Forms of Body Weight Dissatisfaction and Cardiovascular Health-Risk Behaviors

In bivariate analysis, male adolescents dissatisfied feeling underweight were more likely to smoke tobacco and consume alcohol, although the associations were no longer significant after adjusting for sociodemographic characteristics. These associations were also present among adequate weight and dissatisfied feeling overweight, in male adolescents who were more likely to consume alcohol (adjusted odds ratio [aOR]: 1.3; 95% confidence interval [CI]: 1.02–1.76). Female adolescents dissatisfied feeling overweight were more likely to smoke tobacco and consume alcohol, and the association with alcohol use remained significant after adjustment (aOR: 1.4; 95% CI: 1.24–1.70). Girls who were dissatisfied feeling underweight were also more likely to have consumed alcohol in the last 30 days (aOR: 1.2; 95% CI: 1.03–1.45). Underweight girls dissatisfied with their body weight were less likely to smoke tobacco (aOR: 0.2; 95% CI: 0.38–0.74). Adequate weight girls dissatisfied feeling overweight were more likely smoke tobacco (aOR: 1.4; 95% CI: 1.04–1.86) and also to consume alcohol (aOR: 1.5; 95% CI: 1.26–1.73), while adequate weight girls dissatisfied feeling underweight were more likely only to consume alcohol (aOR: 1.3; 95% CI: 1.08–1.54). Moreover, overweight girls dissatisfied feeling overweight were more likely to use alcohol (aOR: 1.5; 95% CI: 1.16–2.06) (Table 3).

Male adolescents dissatisfied feeling overweight were more likely to be inactive and to skip breakfast (aOR: 1.3; 95% CI 1.07–1.54 and aOR: 1.4; 95% CI 1.18–1.56, respectively). These associations were also present among overweight boys dissatisfied feeling overweight (aOR: 1.4; 95% CI 1.07–1.74 and aOR: 1.6; 95% CI 1.28–1.92, respectively), while overweight boys dissatisfied feeling underweight were more likely only to skip breakfast (aOR: 1.9; 95% CI 1.11–3.41). Adequate-weight boys were more likely to skip breakfast (aOR: 1.6; 95% CI 1.30–2.10). Female adolescents dissatisfied feeling underweight and also feeling overweight were more likely to skip breakfast (aOR: 1.2; 95% CI: 1.02–1.30 and aOR: 1.3; 95% CI: 1.27–1.54, respectively). Adequate-weight and overweight girls who were dissatisfied feeling overweight were more likely to skip breakfast (aOR: 1.6; 95% CI: 1.38–1.80 and aOR: 1.3; 95% CI: 1.02–1.61, respectively), but underweight girls dissatisfied feeling underweight were more likely to skip breakfast (aOR: 1.9; 95% CI: 1.14–3.36) (Table 4).

## 4. Discussion

Based on data of a national survey of Brazilian adolescents, the present analyses revealed that at least one of the two forms of BWD was associated with higher odds of cardiovascular health-risk behaviors, and this association was different by gender.

Almost half of adolescents reported BWD, especially dissatisfaction feeling overweight. This result indicated that adolescents believed that they were overweight and/or obese. However, the percentage of adolescents who were overweight and/or obese was lower than the percentage of dissatisfaction feeling overweight [16,17,24]. The same result was found concerning dissatisfaction feeling underweight. Therefore, BWD is more than a physiological indicator as BMI-for-age, since this involves a subjective construct of body. However, it also involves a mutual relationship with weight status, being influenced by it.

BWD was found to be more common amongst female than male adolescents. Gender difference has already been noted in the literature [9,16,17,24,25,26]. Social ideals of unreal beauty are transmitted in the mass media which promote the ideal body to be lean but highly muscular for boys, while for the girls, a slim body [27].

It seems that Brazilian adolescents have been adopting fewer health-risk behaviors than the global surveys show, since, worldwide, 7% of adolescents are tobacco users [28], 26.5% are current alcohol drinkers [29], 80% are inactive [30] and 20% to 40% skipping breakfast [9,31].

The association between BWD and tobacco use remains controversial, as shown in a review that investigated concerns about weight and smoking among adolescents [32]. According to this review, this association will depend in which component or dimension of BWD was considered in the studies [9,10,26]. Furthermore, results should be interpreted considering the rigorous Brazilian legislation towards tobacco purchase and use. Since 2011, in Brazil it has been prohibited to smoke in public places, advertise tobacco in mass media or with misleading advertising and sell it to individuals younger than 18 years [33]. Despite the absence of association between BWD and tobacco use, the dissatisfaction may be a trigger to smoking, because many individuals believe that cigarettes act as a means of weight control or weight loss [34].

In female adolescents, the two forms of BWD were associated with alcohol use. This association has been found elsewhere [9,12,13]. In the Youth Risk Behavior Surveillance (YRBS) study, undertaken in the United States in 2013, female adolescents who reported BWD were more likely to report lifetime alcohol use and heavy episodic drinking [12]. A potential explanation for this association could be the negative parent–adolescent relationships as a risk factor shared for both, BWD and alcohol use [35]. Nevertheless, what we already know is that alcohol use during adolescence can have long-term detrimental effects [2].

In male adolescents, only the dissatisfaction of feeling overweight was associated with physical inactivity. There is no consensus in the literature concerning the association between BWD and physical inactivity. Some studies have shown an association [8,11], but others have not [16,24]. A possible argument is that BWD was measured in different components or dimensions [8,11,16,24]. However, even in studies that used the same methodology when measuring BWD, results were inconsistent. Regarding the dissatisfaction feeling overweight, studies considered did not investigate this specific issue [8,11,16,24].

In female adolescents, the two forms of BWD were associated with skipping breakfast. Given the absence of researches about this association, the interpretation is questionable [14,36,37]. However, the authors are of the view that female adolescents seem to be engaging in this unhealthy eating habit in order to lose weight or to gain weight. It should be noted the importance of breakfast consumption, which reduces the risk of developing type 2 diabetes mellitus [38], plays a protective role in overweight and obesity, and avoids excessive adiposity during adolescence [39].

The theme of body weight dissatisfaction and its relation with unhealthy behaviors is still under development, since the most of the studies have been focusing on eating or mental disorders [37]. However, consistent with the little existing research, the present study demonstrated that body weight dissatisfaction was associated with a range of adolescent health-risk behaviors increasing cardiovascular risk over the life course.

Despite boys and girls being under the influence of the same factors for the development of body weight satisfaction or dissatisfaction [3] (pp. 76–92), the social value internalization is different among genders. The girls generally become more dissatisfied with their bodies looking for a slender body, i.e., dissatisfied feeling overweight [3] (pp. 76–92). On the other hand, boys seem to be divided between those dissatisfied feeling underweight and those dissatisfied feeling overweight [3] (pp. 76–92). Therefore, the assessment of the association between BWD and cardiovascular health-risk behavior should take into account the gendered stratification, as confirmed in the present study.

In the case of the forms of BWD, the hypothesis that having a dissatisfied feeling of underweight or overweight would show different implications for health behaviors was not confirmed. In general, just having BWD, no matter the form, was associated with health-risk behaviors. However, due to scarce studies which assessed these two forms separately [37], this interpretation requires more future studies.

The study reinforces that public policy makers and health professionals should consider adolescents’ thoughts and feelings about their body weight, and not only the assessment of BMI-for-age. Particularly in Brazil, since the National Policy for Children and Adolescents do not consider body weight or body image [40].

This study has some limitations. First, due to the comprehensive nature of this survey, some of the measures of health-risk behaviors were brief and may not provide a deeper exploration of the association with BWD. Second, the cross-sectional design of the study precluded any conclusions about the direction of causality. Third, the data were based on self-reports, and thus, subject to potential memory bias. Fourth, although our analyses controlled for important covariates such adolescent age, BMI-for-age and school type, residual confounding by unmeasured variables is a possibility. Fifth, although ERICA is a nationwide study, only adolescent students of large cities were considered and, therefore, it does not represent those living in towns with populations less than 100,000 inhabitants. Furthermore, ERICA did not include the adolescents who are out of schools. This fact may have caused some impact on the estimates of BWD and behaviors, because these adolescents can be workers or living in a vulnerable socioeconomic condition. However, this study has several strengths that include the national sample of Brazilian adolescents; the assessment of BWD in its two forms; the assessment according to gender; the assessment of the BMI-for-age verified by trained evaluators and not self-reported weight and height; the assessment of health-risk behaviors such as skipping breakfast; and the use of a self-completed form with PDA may have helped to minimize the bias of under- or over-reported behaviors to meet socially acceptable patterns.

First of all, studies should explore the two forms of BWD and to assess the satisfaction as a component of the attitudinal dimension. Additionally, researchers need to move forward and do study interventions to increase BWD among male and female adolescents.

## 5. Conclusions

Adolescents who are dissatisfied with their body weight have higher odds to engage in cardiovascular health-risk behaviors. The findings highlight the need to include body image assessment on adolescents’ health assessment in public health policies.

## Figures and Tables

**Table 1:** Adolescent’s individual and sociodemographic characteristics, body weight dissatisfaction and health-risk behaviors in male and female adolescents.

Variables	Total	Male	Female	*p* ^a^
	n = 71,740	n = 36,013 (50.2%)	n = 35,727 (49.8%)	
	%	%	%	
Age group (years)				0.285
12–14	52.7	52.9	52.5	
15–17	47.3	47.1	47.5	
Skin color				0.182
Nonwhite	60.0	59.4	60.5	
White	40.0	40.6	39.5	
Mother’s schooling (years)				<0.001
<11	44.9	43.3	46.5	
≥11	55.1	56.7	53.5	
Family structure				<0.001
Mother and father	57.3	59.4	55.1	
Only mother	32.4	29.9	35.0	
Only father or no parents	10.3	10.7	9.9	
School type				0.084
Public	82.6	82.0	83.2	
Private	17.4	18.0	16.8	
BMI-for-age status				<0.001
Underweight	2.8	3.5	2.1	
Adequate weight	71.8	70.8	72.7	
Overweight/obesity	25.4	25.7	25.2	
Body weight dissatisfaction				<0.001
Satisfied	55.0	63.6	46.2	
Dissatisfied	45.0	36.4	53.8	
feeling underweight	14.7	14.9	14.5	
feeling overweight	30.3	21.5	39.3	
Tobacco use in the last 30 days				0.127
No	95.2	94.9	95.4	
Yes	4.8	5.1	4.6	
Alcohol use in the last 30 days				0.308
No	77.9	78.4	77.4	
Yes	22.1	21.6	22.6	
Physical inactivity in the last seven days				<0.001
No	51.6	63.0	40.0	
Yes	48.4	37.0	60.0	
Skipping breakfast				<0.001
No	48.4	54.5	42.3	
Yes	51.6	45.5	57.7	

^a^ Chi-square test.

**Table 2:** Adolescent’s individual and sociodemographic characteristics, according to the two forms of body weight dissatisfaction in male and female adolescents.

Variables		Male				Female		
	Satisfied	Dissatisfied Feeling Underweight	Dissatisfied Feeling Overweight	*p* ^a^	Satisfied	Dissatisfied Feeling Underweight	Dissatisfied Feeling Overweight	*p* ^a^
	%	%	%		%	%	%	
Age group (years)				<0.001				<0.001
12–14	66.1	12.0	21.9		48.5	12.9	38.6	
15–17	60.9	18.2	20.9		43.8	16.2	40.0	
Skin color				0.003				<0.001
Nonwhite	64.5	15.4	20.1		47.2	15.2	37.6	
White	62.4	14.2	23.4		44.6	13.5	41.9	
Mother’s schooling (years)				0.031				<0.001
<11	65.3	15.0	19.7		47.5	16.2	36.3	
≥11	61.7	15.4	22.9		42.9	12.9	44.2	
Family structure				0.146				0.099
Mother and father	64.4	14.0	21.6		46.7	13.5	39.8	
Only mother	63.6	15.9	20.5		45.4	15.5	39.1	
Only father or no parents	59.4	17.3	23.3		46.3	16.8	36.9	
School type				<0.001				<0.001
Public	65.2	15.0	19.8		47.4	15.2	37.4	
Private	56.2	14.8	29.0		40.6	11.1	48.3	
BMI-for-age status								
Underweight	50.8	47.2	2.0	<0.001	31.9	66.5	1.6	<0.001
Adequate weight	74.5	17.4	8.1		57.4	16.9	25.7	
Overweight/obesity	35.3	4.0	60.7		15.2	3.1	81.7	

^a^ Chi-square test.

**Table 3:** Association between two forms of body weight dissatisfaction and cardiovascular health-risk behaviors tobacco use and alcohol use in the last 30 days, by gender.

Body Weight Dissatisfaction	Tobacco Use in the Last 30 Days					Alcohol Use in the Last 30 Days				
		Univariate		Multivariate ^a,b^			Univariate		Multivariate ^a,b^	
	%	OR	95% CI	OR	95% CI	%	OR	95% CI	OR	95% CI
**Male adolescents**										
Satisfied	4.7	Ref	-	Ref	-	20.5	Ref	-	Ref	-
Dissatisfied feeling underweight	6.3	1.4 ^c^	1.08–1.73	1.1	0.90–1.47	24.7	1.3 ^c^	1.05–1.53	1.1	0.92–1.35
Dissatisfied feeling overweight	5.3	1.1	0.77–1.65	1.1	0.65–1.97	22.4	1.1	0.95–1.31	0.9	0.75–1.16
Underweight ^e^										
Satisfied	2.5	Ref	-	Ref	-	12.0	Ref	-	Ref	-
Dissatisfied	4.4	1.8	0.62–5.09	1.5	0.49–4.65	19.8	1.7	0.87–3.52	1.5	0.73–3.24
Adequate weight										
Satisfied	4.8	Ref	-	Ref	-	20.8	Ref	-	Ref	-
Dissatisfied feeling underweight	6.6	1.4 ^c^	1.07–1.79	1.2	0.93–1.56	25.9	1.3 ^c^	1.08–1.65	1.1	0.90–1.38
Dissatisfied feeling overweight	6.8	1.4	0.64–3.20	1.4	0.61–3.07	27.2	1.4 ^c^	1.10–1.85	1.3 ^c^	1.02–1.76
Overweight										
Satisfied	4.5	Ref	-	Ref	-	20.9	Ref	-	Ref	-
Dissatisfied feeling underweight	6.3	1.4	0.52–3.94	1.9	0.66–5.30	18.2	0.8	0.45–1.54	1.0	0.55–1.94
Dissatisfied feeling overweight	4.8	1.1	0.66–1.69	0.9	0.57–1.45	20.8	1.0	0.75–1.31	0.9	0.64–1.17
**Female adolescents**										
Satisfied	4.0	Ref	-	Ref	-	19.0	Ref	-	Ref	-
Dissatisfied feeling underweight	4.2	1.0	0.81–1.38	1.0	0.76–1.31	24.0	1.3 ^d^	1.15–1.59	1.2 ^c^	1.03–1.45
Dissatisfied feeling overweight	5.5	1.4 ^c^	1.11–1.80	1.2	0.94–1.66	26.3	1.5 ^d^	1.35–1.71	1.4 ^d^	1.24–1.70
Underweight ^e^										
Satisfied	3.7	Ref	-	Ref	-	16.7	Ref	-	Ref	-
Dissatisfied	0.8	0.2 ^c^	0.05–0.81	0.2^c^	0.38–0.74	15.7	0.9	0.47–1.81	0.8	0.40–1.56
Adequate weight										
Satisfied	3.9	Ref	-	Ref	-	19.3	Ref	-	Ref	-
Dissatisfied feeling underweight	4.7	1.2	0.91–1.59	1.1	0.82–1.47	25.4	1.4 ^d^	1.20–1.68	1.3 ^c^	1.08–1.54
Dissatisfied feeling overweight	5.6	1.5 ^c^	1.01–1.94	1.4^c^	1.04–1.86	27.5	1.6 ^d^	1.37–1.85	1.5 ^d^	1.26–1.73
Overweight										
Satisfied	4.4	Ref	-	Ref	-	15.4	Ref	-	Ref	-
Dissatisfied feeling underweight	2.2	0.5	0.19–1.25	0.5	0.19–1.31	18.1	1.2	0.62–2.38	1.2	0.61–2.53
Dissatisfied feeling overweight	5.4	1.2	0.72–2.17	1.1	0.66–1.90	25.0	1.8 ^d^	1.39–2.42	1.5 ^c^	1.16–2.06

^a^ Multivariate model for gender adjusted for: age, BMI-for-age and school type; ^b^ Multivariate model for gender and BMI-for-age status adjusted for: age and school type; ^c^ *p* < 0.05; ^d^ *p* < 0.001; ^e^ Underweight status was classified into two categories satisfied and dissatisfied, because the category dissatisfied feeling overweight was empty.

**Table 4:** Association between two forms of body weight dissatisfaction and cardiovascular health-risk behaviors physical inactivity in the last seven days and skipping breakfast, by gender.

Body Weight Dissatisfaction	Physical Inactivity in the Last 7 Days					Skipping Breakfast				
		Univariate		Multivariate ^a,b^			Univariate		Multivariate ^a,b^	
	%	OR	95% CI	OR	95% CI	%	OR	95% CI	OR	95% CI
**Male adolescents**										
Satisfied	36.0	Ref	-	Ref	-	43.0	Ref	-	Ref	-
Dissatisfied feeling underweight	37.1	1.0	0.89–1.23	1.0	0.89–1.23	42.1	1.0	0.86–1.10	1.0	0.87–1.11
Dissatisfied feeling overweight	39.7	1.2 ^c^	1.00–1.37	1.3 ^c^	1.07–1.54	55.4	1.6 ^d^	1.48–1.84	1.4 ^d^	1.18–1.56
Underweight										
Satisfied	47.5	Ref	-	Ref	-	34.8	Ref	-	Ref	-
Dissatisfied feeling underweight	51.3	1.2	0.69–1.95	1.1	0.69–1.85	30.2	0.8	0.46–1.43	0.8	0.46–1.33
Dissatisfied feeling overweight	32.9	0.5	0.15–1.92	0.5	0.14–1.78	38.2	1.2	0.29–4.59	1.3	0.34–5.13
Adequate weight										
Satisfied	36.0	Ref	-	Ref	-	43.1	Ref	-	Ref	-
Dissatisfied feeling underweight	35.9	1.0	0.83–1.19	1.0	0.86–1.21	42.3	1.0	0.85–1.10	0.9	0.81–1.04
Dissatisfied feeling overweight	38.4	1.1	0.85–1.45	1.1	0.86–1.47	55.4	1.6^d^	1.31–2.06	1.6 ^d^	1.30–2.10
Overweight										
Satisfied	33.6	Ref	-	Ref	-	43.9	Ref	-	Ref	-
Dissatisfied feeling underweight	27.4	0.7	0.39–1.40	0.7	0.38–1.37	58.2	1.8^c^	1.05–3.00	1.9 ^c^	1.11–3.41
Dissatisfied feeling overweight	40.3	1.3 ^c^	1.04–1.70	1.4 ^c^	1.07–1.74	55.5	1.6^d^	1.31–1.93	1.6 ^d^	1.28–1.92
**Female adolescents**										
Satisfied	61.4	Ref	-	Ref	-	52.7	Ref	-	Ref	-
Dissatisfied feeling underweight	61.6	1.0	0.86–1.18	1.0	0.83–1.15	55.6	1.1 ^c^	1.00–1.25	1.2 ^c^	1.02–1.30
Dissatisfied feeling overweight	57.6	0.8 ^c^	0.79–0.94	1.0	0.83–1.06	64.4	1.6 ^d^	1.44–1.83	1.3 ^d^	1.27–1.54
Underweight										
Satisfied	60.3	Ref	-	Ref	-	36.2	Ref	-	Ref	-
Dissatisfied feeling underweight	69.0	1.5	0.85–2.53	1.5	0.86–2.57	52.9	1.99 ^c^	1.17–3.38	1.9 ^c^	1.14–3.36
Dissatisfied feeling overweight	62.3	1.1	0.30–3.94	1.0	0.30–3.80	53.7	2.04	0.61–6.74	2.2	0.64–7.75
Adequate weight										
Satisfied	61.5	Ref	-	Ref	-	52.4	Ref	-	Ref	-
Dissatisfied feeling underweight	61.0	1.0	0.81–1.18	1.0	0.81–1.18	55.2	1.1 ^c^	1.01–1.26	1.1	0.96–1.21
Dissatisfied feeling overweight	58.7	0.9	0.77–1.03	0.9	0.78–1.04	63.4	1.6 ^d^	1.40–1.81	1.6 ^d^	1.38–1.80
Overweight										
Satisfied	60.5	Ref	-	Ref	-	59.5	Ref	-	Ref	-
Dissatisfied feeling underweight	58.0	0.9	0.51–1.60	0.9	0.51–1.60	66.3	1.3	0.80–2.25	1.3	0.80–2.28
Dissatisfied feeling overweight	56.6	0.8	0.66–1.10	0.8	0.66–1.10	65.3	1.3 ^c^	1.03–1.60	1.3^c^	1.02–1.61

^a^ Multivariate model for gender adjusted for age, BMI-for-age and school type; ^b^ Multivariate model for gender and BMI-for-age status adjusted for age and school type; ^c^ *p* < 0.05; ^d^ *p* < 0.001.

## References

[1] World Health Organization (2014). Global Status Report on Noncommunicable Diseases 2014.

[2] Patton G.C., Sawyer S.M., Santelli J.S., Ross D.A., Afifi R., Allen N.B., Arora M., Azzopardi P., Baldwin W., Bonell C. (2016). Our future: A Lancet commission on adolescent health and wellbeing. Lancet.

[3] Cash T.F. (2012). Body Image: A Handbook of Science, Practice, and Prevention.

[4] Bibiloni M.D.M., Pich J., Pons A., Tur J.A. (2013). Body image and eating patterns among adolescents. BMC Public Health.

[5] Lee W.-T., Kim H.I., Kim J.H., Lee S.-J.R., Hong S., Park E.-C. (2015). Relationships between Body Image, Body Mass Index, and Smoking in Korean Adolescents: Results of a Nationwide Korea Youth Risk Behavior Web-based Survey. Asian Pac. J. Cancer Prev..

[6] Neumark-Sztainer D., Wall M., Story M., Standish A.R. (2012). Dieting and Unhealthy Weight Control Behaviors during Adolescence: Associations with 10-Year Changes in Body Mass Index. J. Adolesc. Health.

[7] World Health Organization (2016). Growing up Unequal: Gender and Socioeconomic Differences in Young People’s Health and Well-Being. Health Behaviour in School-Aged Children (HBSC) Study: International Report from the 2013/2014.

[8] Finne E., Bucksch J., Lampert T., Kolip P. (2011). Age, puberty, body dissatisfaction, and physical activity decline in adolescents. Results of the German Health Interview and Examination Survey (KiGGS). Int. J. Behav. Nutr. Phys. Act..

[9] Martini M.C.S., De Assumpção D., Barros M.B.D.A., Canesqui A.M., Filho A.D.A.B. (2016). Are normal-weight adolescents satisfied with their weight?. Sao Paulo Med. J..

[10] Pénzes M., Czeglédi E., Balázs P., Foley K.L. (2012). Factors Associated with Tobacco Smoking and the Belief about Weight Control Effect of Smoking among Hungarian Adolescents. Central Eur. J. Public Health.

[11] Sampasa-Kanyinga H., Hamilton H., Willmore J., Chaput J.-P. (2017). Perceptions and attitudes about body weight and adherence to the physical activity recommendation among adolescents: The moderating role of body mass index. Public Health.

[12] Schlissel A.C., Schwartz T.T., Skeer M.R. (2017). The Association between Body Image and Behavioral Misperception (BIBM) and Alcohol Use among High School Girls: Results from the 2013 Youth Risk Behavioral Survey. J. Stud. Alcohol Drugs.

[13] Xie B., Chou C.-P., Spruijt-Metz D., Reynolds K., Clark F., Palmer P.H., Gallaher P., Sun P., Guo Q., Johnson C.A. (2006). Weight perception and weight-related sociocultural and behavioral factors in Chinese adolescents. Prev. Med..

[14] Claro A.P.R.A., Santos M.A.S., Oliveira-Campos M. (2014). Body image and extreme attitudes toward weight in Brazilian schoolchildren (PeNSE 2012). Rev. Bras. Epidemiol..

[15] Moehlecke M., Blume C.A., Cureau F.V., Kieling C., Schaan B.D. (2018). Self-perceived body image, dissatisfaction with body weight and nutritional status of Brazilian adolescents: A nationwide study. J. Pediatr..

[16] Christofaro D.G., Ritti-Dias R.M., De Andrade S.M., De Moraes A.C., Cabrera M.A., Fernandes R.A. (2015). Body weight dissatisfaction and its correlates among Brazilian adolescents. Med. Express.

[17] Santos E.M.C., Tassitano R.M., Nascimento W.M.F.D., Petribu M.D.M.V., Cabral P.C. (2011). Satisfação com o peso corporal e fatores associados em estudantes do ensino médio. Rev. Paul. Pediatr..

[18] Instituto Brasileiro de Geografia e Estatística (2016). Pesquisa Nacional de Saúde do Escolar 2015.

[19] Bloch K.V., Szklo M., Kuschnir M.C.C., Abreu G.D.A., Barufaldi L.A., Klein C.H., De Vasconcelos M.T.L., Veiga J.P.C.B., Figueiredo V.C., Dias A. (2015). The study of cardiovascular risk in adolescents—ERICA: Rationale, design and sample characteristics of a national survey examining cardiovascular risk factor profile in Brazilian adolescents. BMC Public Health.

[20] World Health Organization (2017). WHO Recommendations on Adolescent Health: Guidelines Approved by the WHO Guidelines Review Committee.

[21] Sallis J.F., Strikmiller P.K., Harsha D.W., Feldman H.A., Ehlinger S., Stone E.J., Williston J., Woods S. (1996). Validation of interviewer- and self- administered physical activity checklists for fifth grade students. Med. Sci. Sports Exerc..

[22] De Onis M., Onyango A.W., Borghi E., Siyam A., Nishida C., Siekmann J. (2007). Development of a WHO growth reference for school-aged children and adolescents. Bull. World Health Organ..

[23] Organização para a Cooperação e Desenvolvimento Econômico (2016). Brasil no PISA 2015: Análises e Reflexões Sobre o Desempenho dos Estudantes Brasileiros.

[24] Al Sabbah H., Vereecken C., Elgar F.J., Nansel T.R., Aasvee K., Abdeen Z., Ojala K., Ahluwalia N., Maes L. (2009). Body weight dissatisfaction and communication with parents among adolescents in 24 countries: International cross-sectional survey. BMC Public Health.

[25] Calzo J.P., Sonneville K.R., Haines J., Blood E.A., Field A.E., Austin S.B. (2012). The Development of Associations among Body Mass Index, Body Dissatisfaction, and Weight and Shape Concern in Adolescent Boys and Girls. J. Adolesc. Health.

[26] Del Duca G.F., Garcia L.M.T., de Sousa T.F., Oliveira E.S.A., Nahas M.V. (2010). Body weight dissatisfaction and associated factors among adolescents. Rev. Paul. Pediatr..

[27] Reel J.J., Voelker D.K., Greenleaf C. (2015). Weight status and body image perceptions in adolescents: Current perspectives. Adolesc. Health Med. Ther..

[28] World Health Organization (2018). WHO Global Report on Trends in Prevalence of Tobacco Smoking 2000–2025.

[29] World Health Organization (2018). Global Status Report on Alcohol and Health 2018.

[30] Sallis J.F., Bull F., Guthold R., Heath G.W., Inoue S., Kelly P., Oyeyemi A.L., Perez L.G., Richards J., Hallal P.C. (2016). Progress in physical activity over the Olympic quadrennium. Lancet.

[31] Underwood J.M., Brener N., Thornton J., Harris W.A., Bryan L.N., Shanklin S., Deputy N., Roberts A.M., Queen B., Chyen D. (2020). Youth Risk Behavior Surveillance—United States, 2019.

[32] Potter B., Pederson L.L., Chan S.S.H., Aubut J.-A.L., Koval J.J. (2004). Does a relationship exist between body weight, concerns about weight, and smoking among adolescents? An integration of the literature with an emphasis on gender. Nicotine Tob. Res..

[33] Levy D., De Almeida L.M., Szklo A. (2012). The Brazil SimSmoke Policy Simulation Model: The Effect of Strong Tobacco Control Policies on Smoking Prevalence and Smoking-Attributable Deaths in a Middle Income Nation. PLoS Med..

[34] Bašková M., Holubčíková J., Baška T. (2017). Body-Image Dissatisfaction and Weight-Control Behaviour in Slovak Adolescents. Cent. Eur. J. Public Health.

[35] Visser L., De Winter A.F., Reijneveld S.A. (2012). The parent–child relationship and adolescent alcohol use: A systematic review of longitudinal studies. BMC Public Health.

[36] Córdoba Caro L.G., Luengo Pérez L.M., Feu S., Preciado V.G. (2015). Satisfaction with weight and characteristics of eating disorders in high school. An. Pediatr..

[37] Duarte L.S., Palombo C.N.T., Solis-Cordero K., Kurihayashi A.Y., Steen M., Borges A.L.V., Fujimori E. (2020). The association between body weight dissatisfaction with unhealthy eating behaviors and lack of physical activity in adolescents: A systematic review. J. Child Health Care.

[38] Bi H., Gan Y., Yang C., Chen Y., Tong X., Lu Z. (2015). Breakfast skipping and the risk of type 2 diabetes: A meta-analysis of observational studies. Public Health Nutr..

[39] Blondin S.A., Anzman-Frasca S., Djang H.C., Economos C.D. (2016). Breakfast consumption and adiposity among children and adolescents: An updated review of the literature. Pediatr. Obes..

[40] Brasil Ministério da Saúde (2018). Política Nacional de Atenção Integral à Saúde da Criança: Orientações para implementação.

